# Assessment of net lending strategy to better reach mobile and migrant populations in malaria endemic areas of Cambodia

**DOI:** 10.1186/s40249-018-0489-1

**Published:** 2018-10-18

**Authors:** Dysoley Lek, Deyer Gopinath, Sovann Ek, Sopheab Heng, Sreng Bun, Chy Say, Nguon Sokomar, Kheang Soy Ty, Huy Rekol

**Affiliations:** 1 National Center for Parasitology Entomology and Malaria Control, Corner St. 92, village Trapeng Svay, Phnom Penh Thmey, Sen Sok, Phnom Penh, Cambodia; 2 School of Public Health, National Institute of Public Health, Phnom Penh, Cambodia; 3 World Health Organization Country Office for Thailand, Nonthaburi, Thailand; 4 University Research Co, LLC, Phnom Penh, Cambodia; 5 Department of Communicable Diseases Control, Ministry of Health, Phnom Penh, Cambodia

**Keywords:** Cambodia, Malaria, Mobile and migrant population, Net, Lending

## Abstract

**Background:**

In Cambodia, internal migration involves migrants moving from non-malaria endemic areas to malaria endemic areas and vice versa. The majority of them work in farms or forests with various malaria transmission levels. In Cambodia, as one of the national approaches to ensure LLIN accessibility and use among mobile and migrant populations (MMPs), a lending scheme of long lasting insecticide treated nets (LLINs) was initiated among farm workers. Through this net lending program, LLINs and long-lasting insecticide treated hammock nets (LLIHNs) will be distributed annually at workplace (e.g. longstanding farms, plantations, industrial sites, as identified by operational district and health center staff) on a ratio of one LLIN per one worker. The main objective of this study is to assess MMPs’ accessibility to LLINs through a lending scheme with plantation owners in remote malaria endemic areas of Cambodia.

**Methods:**

The study used a cross-sectional survey among MMPs using two-stage cluster sampling method. The sampling frame is the list of farms in the four provinces of Banteay Meanchey, Battambang, Pailin, and Pursat in western and northwestern Cambodia bordering with Thailand where the LLIN lending scheme was implemented and where an estimated 100 000 MMPs worked annually. The assessment was carried out from January to February 2013 in these four provinces. It was estimated that 768 workers would be required.

**Results:**

A total of 702 MMPs were interviewed. The ratio of male: female is 1:1. The age group of 21–60 was the largest accounting for 77.6%. About 91% of the MMPs stayed on the farm for less than 6 months. 93.2% of them owned either untreated or insecticide treated nets. LLINs and LLIHNs accounted for 89.5%; and 46.6% of them borrowed the nets from a lending scheme. Among those workers who have LLINs/LLIHNs, 99% slept under LLINs/LLIHNs the night before. However, only 87.4% knew that sleeping under LLINs/LLIHNs protects against malaria.

**Conclusions:**

LLIN lending scheme provides an important delivery channel for a substantial percentage of net accessibility (46.6%) to the Cambodian national free-net distribution campaign in remote malaria endemic areas.

**Electronic supplementary material:**

The online version of this article (10.1186/s40249-018-0489-1) contains supplementary material, which is available to authorized users.

## Multilingual abstracts

Please see Additional file 1 for translations of the abstract in to the five official working languages of the United Nations

## Background

The “mass effect” of a high coverage with insecticide treated nets (ITNs) in the general population, commonly done as an annual campaign by most malaria programs, leads to an overall reduction in malaria transmission, enhancing protection to both net users and non-users [1–3]. With the introduction of long lasting insecticide treated nets (LLIN), net durability or average ‘lifespan’ according to World Health Organization (WHO) (2011), is on the average, up to three years. The rate of replacement in continuous distribution systems and the appropriate interval between campaigns depend on the characteristics of the human population, malaria transmission levels and immune status of the population [4]. However, the provision of ITNs to defined high-risk groups especially mobile and migrant populations (MMPs) remains a challenge [5].

While tremendous strides have been made toward eliminating malaria in Cambodia [6], pockets of risk persist in border areas that are remote and have high population mobility. A census in 2010 [7] estimates that the migrant population represents about 14% of the total population in Cambodia especially spread over in border provinces with Thailand in the north-western provinces. These populations often travel between endemic and non-endemic areas, mobility driven by job opportunities created by economic development [8, 9]. The 2009 Cambodia Containment Survey reported that the prevalence of malaria among mobile populations is significantly higher than the national rates of the general population with ‘forest-goers’, a sub-group of the MMP population, reported to have the highest malaria prevalence rates of 4.1% by microscopy and 11.4% by polymerase chain reaction [10].

The rapid development of cassava, corn and fruit orchards in the forest and forest-fringe areas of the north, west and northeast, and in the rubber plantations of the east and northeast has contributed to favorable conditions for local malaria vectors where migrant workers are thought to contribute to the seasonal transmission of malaria [11]. A national survey to map private and family-run plantations in Cambodia carried out by Population Services International (PSI) in 2013 [12], showed that in 17 out of 20 malaria endemic provinces, there were 87 720 workers employed in 2012 and that majority (82%) of the plantations were family owned with almost half of these by Cambodian nationals with the remaining by Vietnamese and Chinese and were predominantly rubber plantations.

In Cambodia, as per national malaria policy, ITN/LLIN were distributed free of charge to all age groups since 2000 [6]. Within a ‘four pillar’ framework [13] with the objective of increase coverage of impregnated mosquito nets, diagnosis and treatment of malaria, the Cambodian National Malaria Program (CNM) initiated several public-private partnerships with special focus on mobile migrant workers engaged in the private health sector (private clinics, private pharmacies) and in the private non-health sector such taxi drivers, mosquito net sellers and farm owners. Cambodia’s National Strategic Plan for Elimination of Malaria 2011–2015 called for one LLIN per person and one long-lasting insecticide treated hammock net (LLIHN) per family provided for free to those living in malaria endemic villages. This included the retreatment of existing conventional nets with long-lasting insecticide. Nearly three million people, living less than 2 km from the forest edge, are targeted for LLINs and LLIHN. Mobile and migrant populations irrespective of nationality will receive one LLIN distributed either free or on loan from large-scale employers.

In 2012, an estimated 3.2 million people were living in malaria endemic areas. The coverage of bed nets in 2012 was only 56% (1.8 million people), with 1 881 594 LLINs and 268 700 hammock nets and 72 946 re-impregnated nets distributed during 2012 [CNM, 2013].

Previous surveys and reports as shown in Table 1 have documented LLIN access within the general and mobile populations in Cambodia. In recent years, mechanisms to make LLINs accessible for MMPs such as a net lending scheme, forest goers’ packages, “touch points”, have been implemented (Table 2) by the national malaria program in collaboration with non-governmental organizations (NGOs).

**Table 1:** Recent surveys and reports documenting LLIN access within the general and mobile populations in Cambodia

Survey	Salient findings
Achievements of National Malaria Control Program, 2012^1^	• The LLIN coverage in farms was through a strategy of distributing the nets to farm owners through a net loaning scheme. In 2012, a reported 2109 farm owners employing 15 768 migrants received 244 678 LLINs and 5044 hammock nets • 99.7% of households reporting having at least one net of any type • 77.8% of households having at least one insecticide-treated net • 53% of households in at risk areas had sufficient nets (one net per two people)
Cambodian malaria survey in 2013^2^	• 49.9% reported sleeping under an insecticide-treated net the previous night • 57.3%, living in less than 2 km from the forest reporting sleeping under an LLIN the previous night • 43% of people who slept overnight in the forest in the previous six months reporting using an ITN on their last trip to the forest
Survey by PSI Cambodia in 2013, in plantations in 17 malaria endemic provinces	• 40% had experience with net lending schemes • Over 70% of all workers come with their families while very few enterprises (20%) had health services for their workers on site. Of those with on-site health facilities, only 22% and 34% respectively had malaria testing and treatment services available^3^.
KAP Survey by PFD, 2014	• 69% (20% ITN, 46% LLIN, 3% treated hammock net) of plantation and company workers in Koh Kong and Kratie provinces slept under a treated net the previous night

*LLIN* Long lasting insecticide treated net, *ITN* Insecticide treated net, *PSI* Population Services International, *KAP* Knowledge attitude and practice, *PFD* Partners for Development

^1^Achievements of National Malaria Control Program 2012. Annual Conference of National Center for Malaria Control, Parasitology and Entomology . 21–22 Mar 2013, NAGA WORLD Hotel, Phnom Penh. Presentation by: Dr. Po Ly, National Centre for Malaria Control, Parasitology and Entomology, (CNM), Cambodia. http://www.cnm.gov.kh/userfiles/file/Annual%20Report%202012/1_CNM_Presentation.pdf

^2^http://www.malariaconsortium.org/resources/publications/624/cambodia-malaria-survey-2013 . Publication date: 1st August 2015

^3^PSI Cambodia. The PPM Program & Private Plantations Preliminary data. Slide Presentation at the National Malaria Drug Policy Meeting. Phnom Penh: Cambodia; 30–31 January 2014

**Table 2:** Strategies to improve LLIN access to mobile and migrant populations in Cambodia

Strategy	Year initiated	Target areas	Provinces	Target group	Channel of delivery
Bed net lending scheme	2009	5 operational districts in 4 provinces	Banteay Meanchey, Battambang, Pailin, and Pursat	Plantation/farm workers	Engagement with farm owners
Touch Points for bed net distribution	2013	1 operational district	Steung Treng	Sites of primary contact with MMPs - mainly forest goers, miners in remote areas	Small grocery shops, boat port, middlemen or broker where MMPs gather for personal belongings or sell their forest products before re-entering into the forest.
“Forest package” project	2013	2 operational district	Kratie and Koh Kong	Distributes subsidized backpacks equipped with insect repellent, LLIN/LLIHs, flashlight and an informative brochure on malaria to forest-goers	Through a voucher system between the trained mobile and plantation malaria workers (MMW/PMW), local retailers and the forest goers. The MMW/PMW collects and transports blood samples, test and provide treatment for patients testing positive for malaria
Bed net topping up scheme	2014	5 operational districts in 4 provinces	Battambang, Pursat, Oddarmeanchey, Banteay Meanchey	MMPs including forest goers and local residents who plan to travel to the forest (LLIHN). Topping up scheme also replaced damaged LLIHN among local residents.	Through village malaria workers

*LLIN* Long lasting insecticide treated net, *LLIHN* Long lasting insecticide treated hammock net, *MMP* Mobile and migrant population, *MMW* Mobile malaria worker, *PMW* Plantation malaria worker

The LLIN lending scheme was pioneered in Cambodia during the Malaria Control in Cambodia (MCC) Project, implemented by University Research Co. LLC (URC) in collaboration with CNM. Under the guidance and coordination of the Provincial Health Department (PHD), operational district and health center, the lending scheme distributes bed nets to MMPs who travel to, and live or work in farms or private companies within malaria endemic area for short periods of time (less than six months) [14]. Farm owners loan bed nets to the workers and collect them when the workers leave. As worker turnover is high, this arrangement reduces the number of bed nets needed at the farms. Workers coming from provinces where malaria is not endemic, were not fully aware of the risks of getting malaria in the farms. Many did not bring mosquito nets with them and then fell sick [15]*.* As part of the efforts to reach mobile MMPs, URC initiated a pilot implementation of the bed net lending scheme in 2009 in Sampov Loun operational district. A total of 20 443 LLINs were distributed to 1567 family-sized farms in 13 villages. Before implementing the scheme, health center staff and the URC project team conducted a census among households in these villages to estimate the number of local and MMP workers based on their type or nature of their work. This information was crosschecked and verified by the village chief/ village malaria workers (VMWs). Households with MMP workers engaged in farming activities were selected to receive LLINs/LLIHNs through the scheme. Eligible farm owners were notified in advance, briefed about the lending scheme and committing their participation. Malaria health education was also provided before the owners could receive the nets. The owners were asked to manage the distribution by lending out LLIN/LLIHNs to their existing or newly recruited workers and to collect them back when their workers completed or left their work. The owners may request additional nets from the nearest VMW if they need more nets for their workers. A buffer system was implemented at two levels to ensure adequate stocks of nets. At the village level, VMWs were given a buffer stock of 50 LLIHNs and 50 LLINs. At the health center, a buffer stock of 100 LLIHNs and 100 LLINs Net were maintained. Additional nets could also be requested from the operational district after the VMW verifies the actual needs on site at the farm and makes a formal request. The VMW reports or updates the number of new farms, number of workers, nets distributed, and the buffer balance of nets to the health facility staff and URC team during monthly VMW meetings at the health center.

In 2011, URC conducted an internal qualitative assessment on the feasibility of the lending scheme and the acceptability among the farm owners in Sampov Loun and Pailin operational district. The finding suggested that the lending scheme had been well accepted but could be implemented in a more participatory manner. The lending scheme was then scaled up to an additional 8 operational districts in 2012 over July to October.

The main objective of this study is to assess MMPs’ accessibility to LLINs through a net lending scheme with plantation owners to reach MMP workers in remote malaria endemic areas of Cambodia.

## Methods

### Study design and study area

This was a cross-sectional study among farm workers in the lending scheme villages. The assessment was carried out in four provinces of Banteay Meanchey, Battambang, Pailin, and Pursat in western and northwestern Cambodia bordering with Thailand, where an estimated 100 000 MMPs worked annually. The LLIN lending scheme was scaled up by the URC Control and Prevention of Malaria Project (CAP-Malaria) in 5 out of 8 operational districts of these four provinces and represented about 90% of the total farms in the 4 provinces.

### Study population and study period

Migrant workers and farm owners who worked in small to big sized farms in the 4 provinces of Banteay Meanchey, Battambang, Pailin, and Pursat. The assessment was carried out from January to February 2013.

### Sample size determination

The sample size was calculated using a sample single proportion with the prevalence of utilization of LLIN among farm workers estimated to be 50%. With 95% confidence interval and margin of error of 5%, a sample size of 384 farm workers was obtained. As it was likely that some of farm workers in selected farms were not going to be available for the interview due to their mobility, thus a design effect of 2 was used. The total sample would be 768 workers.

### The sampling method

The sampling frame was developed on Microsoft Excel format by URC with a list of the all farms in each of the 8 operational districts with estimated number of farm workers. Two-stage cluster sampling approach was used to select the MMPs from the list. Clusters were the farm unit. In the first stage, we used probability proportional to the farm size to select 150 clusters from four provinces (Banteay Meanchey = 27, Battambang = 72, Pailin = 42, and Pursat = 9). At the second stage, we randomly selected five workers in each selected cluster (farm unit).

### Data collection method and tools

Workers were interviewed at their working places. In farms that had more than five workers, the interview team randomly selected five workers for the interview, while in farms with five or less workers, all the workers were interviewed. If the selected farms were closed on the interview date, the team replaced them with nearest farms in the sample frame.

### Questionnaire survey

A questionnaire was designed to collect data from the workers in order to assess the coverage, utilization, and knowledge of LLIN use among the migrant workers. The questionnaire was developed based on the objectives and was subsequently pre-tested by an external consultant for this assessment along with the URC team before the field work. After the pretest, no major changes in questionnaire were made, except simplification of questions for better understanding among the interviewees. The interviewers were health center and operational district staff selected from the respective operational districts. They underwent practical trainings on the questionnaire and interview process before the actual field work data collection. A standard operating procedure was developed as a guide for the interviewers. During data collection, interviewers were divided into groups, led by one URC supervisor.

### Data management and statistical methods

Quantitative data were first checked manually for completeness and then double-entered and validated in Epi-Data version 3.1(http://www.epidata.dk/). Epi Info 2000 (Division of Health Informatics & Surveillance, Center for Surveillance, Epidemiology & Laboratory Services, US CDC, Atlanta, USA) was used for data processing and analysis. The key variables to be assessed includied (1) proportion of farm workers who have temporarily or permanently own one LLIN/LLIHNs available for use at the time of interview, and (2) proportion of farm workers who reported to have slept under LLIN/ITN in the night preceding the survey. Descriptive statistics were used to describe the data in terms of proportion, frequency for categorical variables. Mean, median and range were calculated for continuous variables. Chi-squared (*χ*^2^) test was used to compare proportions 5% (*P* < 0.05) level of significance to examine the relation between bed net ownership, use and willingness to pay.

### Ethical consideration

There was no major ethical issue related to this survey. The study was approved by the National Ethics Committee for Health Research in Cambodia on 26 August 2013 with referring letter of number 0159. The confidentiality of the participants was ensured by using codes instead of actual names on the questionnaires. Prior to the interview, a verbal informed consent was obtained from all participants. Prior notice of the study team’s visit was also made to inform all stakeholders.

## Results

### Farm workers place of origin and demographics

**Fig. 1:**
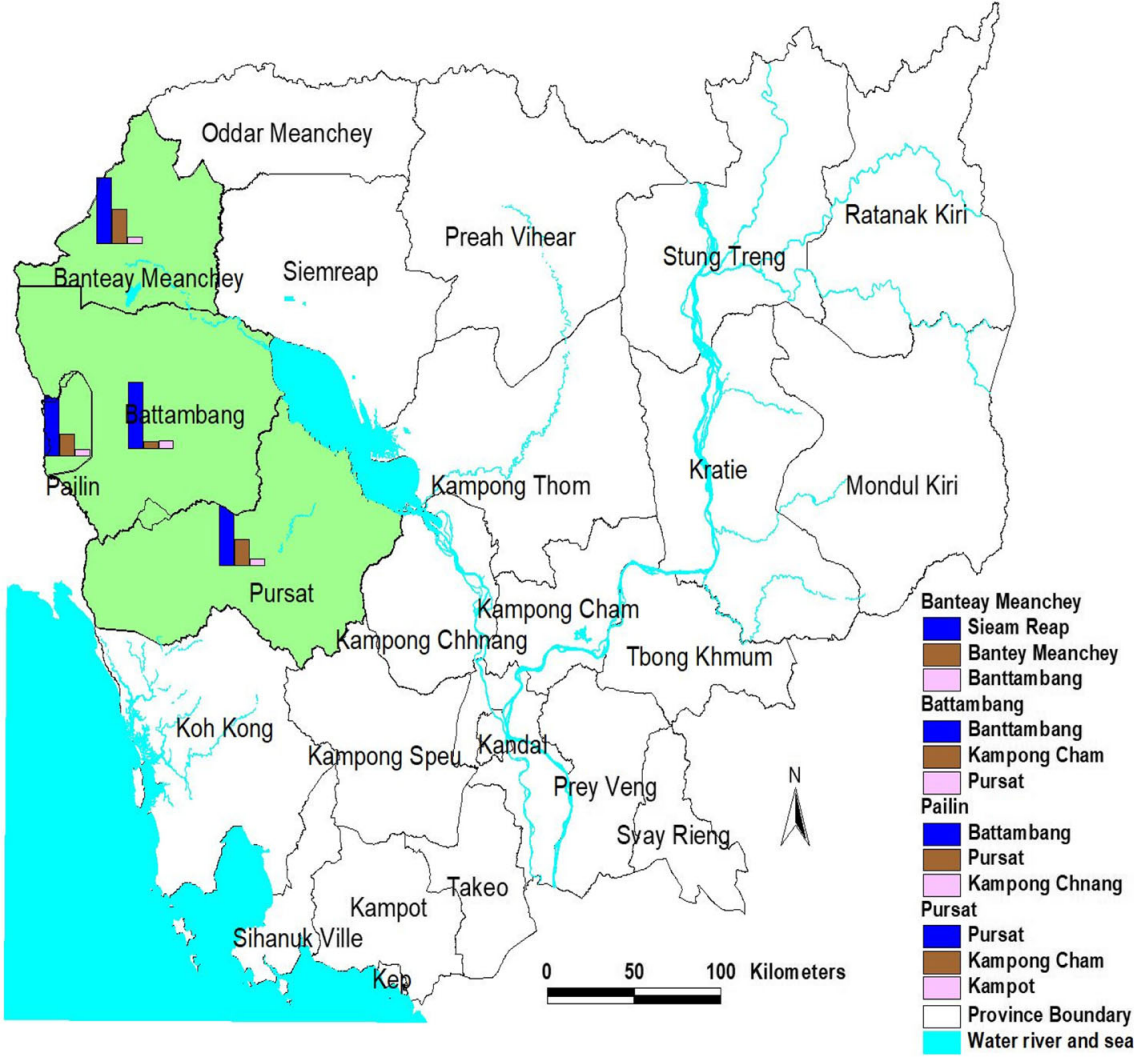
Survey sites: Workers by province of origin

In total, 153 farm owners (farms) and 702 farm workers were interviewed in the four provinces of Battambang, Pursat, Pailin, Bantey Meanchey, under the catchment of 28 health centers (Fig. 1). The intended sample size of 768 workers could not be met because the MMP workers had completed their work and moved out from the farms. Majority of MMP workers originated from the western provinces. About 56% of the respondents were from Battambang province because the majority of farms were located there (Table 3 and Fig. 1). 66.2% of the respondents came from malaria endemic areas where 33.8% from non-endemic areas. Endemic areas refer to area where malaria transmission occurs and net distribution is required. At the time of this survey 47 operational districts were classified as endemic in 21 of the 25 provinces in Cambodia.

**Table 3:** Farm workers by gender and province of origin by region

Province of origin	Male		Female		Total	
	*n*	%	*n*	%	*n*	%
Western region (Provinces: Battambang, Pursat, Banteay Meanchey, Pailin)	222	31.6	244	34.8	466	66.4
Northwest region (Provinces: Oddar Meanchey, Siem Reap, Preah Vihear, Kampong Thom)	37	5.3	24	3.4	61	8.7
North eastern region (Provinces: Kampong Cham, Kratié, Stung Treng)	14	2.0	23	3.3	37	5.3
Southern region (Provinces: Kampong Chhnang, Kampong Speu, Koh Kong, Takéo, Kampot)	54	7.7	43	6.1	97	13.8
Eastern region (Provinces: Kandal, Phnom Penh Municipality, Prey Veng, Svay Rieng)	21	3.0	20	2.8	41	5.8
Total	348	49.6	354	50.4	702	100

Male and female workers (49.6% and 50.4% respectively) were equally distributed in each of the four provinces of which 7% of the female respondents reported to be pregnant at the time of the survey (Table 4). The majority of the respondents (77.6%) were from 21 to 60 years old with a mean age of 33 years. However, 9% of them were aged below 18 years and 4% were elderly aged 60 years or above. 67.8% of the respondents were married. Among those married, 98.8% (471/476) travelled to the farms with their family members. Before coming to work in the current farm, over half of them had experience of working in other farm(s) while less than one third (*n* = 193) at some point had crossed national border to work in a neighboring country. 87.4% of respondents knew that sleeping under LLINs/LLIHNs protected them from getting malaria while 77.2% have low (58.1% primary school level only) or no education at all (19.1% no formal education). The most common form of transportation used by MMPs to travel from their place of origin to the farms was by taxis (74.5%). 91% of the respondents stayed less than 6 months in the farms with 58% (342/593) stayed and worked in the farm for less than 15 days, 78% (463/593) for less than 30 days, 91% (540/593) less than 60 days, and 9% (53/593) for over 60 days or more.

**Table 4:** Characteristics of farm workers interviewed in four provinces

		Province									
		Bantey Meanchey		Battambang		Pursat		Pailin		Total	
		*n*	%	*n*	%	*n*	%	*n*	%	*n*	%
Gender											
Male		50	51.5%	183	46.8%	11	55.0%	104	53.6%	348	49.6%
Female		47	48.5%	208	53.2%	9	45.0%	90	46.4%	354	50.4%
*Total*		97	100%	391	100%	20	100%	194	100%		
Age (years)											
< 20		17	17.5%	81	20.7%	3	15.0%	31	16.0%	132	18.8%
21–40		57	58.8%	191	48.8%	10	50.0%	124	63.9%	382	54.4%
41–60		22	22.7%	102	26.1%	6	30.0%	33	17.0%	163	23.2%
> 60		1	1.0%	17	4.3%	1	5.0%	6	3.1%	25	3.6%
*Total*		97	100%	391	100%	20	100%	194	100%		
Marital Status											
Married		69	71.1%	264	67.5%	14	70.0%	129	66.5%	476	67.8%
Single		24	24.7%	105	26.9%	4	20.0%	61	31.4%	194	27.6%
Divorced/Widowed		4	4.1%	22	5.6%	2	10.0%	4	2.1%	32	4.6%
*Total*		97	100%	391	100%	20	100%	194	100%		
Education											
No education		21	21.6%	84	21.5%	3	15.0%	26	13.4%	134	19.1%
Primary		57	58.8%	214	54.7%	13	65.0%	124	63.9%	408	58.1%
Secondary or higher		19	19.6%	93	23.8%	4	20.0%	44	22.7%	160	22.8%
*Total*		97	100%	391	100%	20	100%	194	100%		
Means of transportation to the farms											
Taxi		87	89.7%	273	69.8%	17	85.0%	146	75.3%	523	74.5%
Bus		0	0.0%	24	6.1%	1	5.0%	26	13.4%	51	7.3%
Moto Taxi		4	4.1%	27	6.9%	1	5.0%	5	2.6%	37	5.3%
Transportation provided by farm owner		0	0.0%	6	1.5%		0.0%	16	8.2%	22	3.1%
Other Means		6	6.2%	61	15.6%	1	5.0%	1	0.5%	69	9.8%
*Total*		97	100%	391	100%	20	100%	194	100%		
Came alone or with family											
Alone		36	37.1%	111	28.4%	11	55.0%	73	37.6%	231	32.9%
With Family		61	62.9%	280	71.6%	9	45.0%	121	62.4%	471	67.1%
*Total*		97	100%	391	100%	20	100%	194	100%		
Length of stay in the farms											
Less than 6 months	Male	49	50.5%	164	41.9%	9	45.0%	97	50.0%	319	45.4%
	Female	43	44.3%	189	48.3%	8	40.0%	79	40.7%	319	45.4%
Over 6 months	Male	1	1.0%	19	4.9%	2	10.0%	7	3.6%	29	4.1%
	Female	4	4.1%	19	4.9%	1	5.0%	11	5.7%	35	5.0%
*Total*		97	100%	391	100%	20	100%	194	100%		
Work permanently or seasonally											
Permanent	Male	0	0.0%	38	9.7%	1	5.0%	11	5.7%	50	7.1%
	Female	4	4.1%	37	9.5%	1	5.0%	17	8.8%	59	8.4%
Seasonal	Male	50	51.5%	145	37.1%	10	50.0%	93	47.9%	298	42.5%
*Total*	Female	43	44.3%	171	43.7%	8	40.0%	73	37.6%	295	42.0%
		97	100%	391	100%	20	100%	194	100%		
Experience of crossing international border											
Never	Male	25	25.8%	130	33.2%	9	45.0%	69	35.6%	233	33.2%
	Female	23	23.7%	178	45.5%	8	40.0%	67	34.5%	276	39.3%
Cross to Thailand	Male	25	25.8%	53	13.6%	2	10.0%	35	18.0%	115	16.4%
	Female	24	24.7%	30	7.7%	1	5.0%	23	11.9%	78	11.1%
Cross to Vietnam	Male	0	0.0%	0	0.0%	0	0.0%	0	0.0%	0	0.0%
	Female	0	0.0%	0	0.0%	0	0.0%	0	0.0%	0	0.0%
*Total*		97	100%	391	100%	20	100%	194	100%		

### Bed net ownership among farm workers

93% of the interviewed farm workers reported to own a mosquito net, either LLIN, conventional untreated bed net or LLIHN (Table 5). 89.5% (628/702) of them owned LLINs/LLIHNs while working in the farm/plantation where they were interviewed. The proportion of net ownership (any types) among male (92%) and female (95%) is not significantly different. There was no significant difference between male and female ownership of any type of nets although women owned slightly more LLINs/LLIHNs than men (47.7% vs 42.2%). Almost half of the respondents borrowed nets from the farm owners (46.6%). 32.9% used the nets taken from home of origin while 13.5% bought in the local markets. 99% of the respondents reported to have slept under a mosquito net in the night preceding the survey, while 94% of them did so in all the last three nights and 92% always slept under the mosquito net since arrival in the farm. There was no observed difference between males and females across the four provinces with female and male workers reported to have slept under any types of nets last night equally the same (*P =* 0.31). Among workers that owned an ITN (*n* = 628), majority (87.4%) understood the benefits of using a treated bet net. Nearly 94% of the total interviewed reported to have gone to bed before 10:00 pm.

**Table 5:** Ownership and use of bed nets in the four provinces

		Province									
		Bantey Meanchey		Battambang		Pursat		Pailin		Total	
		*n*	%	*n*	%	*n*	%	*n*	%	*n*	%
Bed net ownership (among all workers, *n =* 702)											
Having a bed net (*n =* 654)											
LLIN	Male	46	47.40%	146	37.30%	11	55.00%	80	41.20%	283	40.30%
	Female	47	48.50%	188	48.10%	9	45.00%	85	43.80%	329	46.90%
LLIHN	Male	3	3.10%	4	1.00%	0	0.00%	6	3.10%	13	1.90%
	Female	0	0.00%	3	0.80%	0	0.00%	0	0.00%	3	0.40%
Conventional untreated bed net	Male	0	0.00%	0	0.00%	0	0.00%	0	0.00%	0	0.00%
	Female	0	0.00%	0	0.00%	0	0.00%	0	0.00%	0	0.00%
Conventional untreated hammock	Male	1	1.00%	12	3.10%	0	0.00%	10	5.20%	23	3.30%
	Female	0	0.00%	2	0.50%	0	0.00%	1	0.50%	3	0.40%
***Total***		**97**		**355**		**20**		**182**		**654**	**93.20%**
No bed net (*n =* 48)											
	Male	0	0.00%	21	5.40%	0	0.00%	8	4.10%	29	4.10%
	Female	0	0.00%	15	3.80%	0	0.00%	4	2.10%	19	2.70%
Source of nets (any net), (*n =* 654)											
Lending scheme		69	71.00%	173	49.00%	15	75.00%	48	26.00%	305	46.60%
Bought		0	0.00%	57	16.00%	0	0.00%	31	17.00%	88	13.50%
Existing nets		26	27.00%	106	30.00%	3	15.00%	80	44.00%	215	32.90%
Others		2	2.00%	19	5.00%	2	10.00%	23	13.00%	46	7.00%
***Total***		**97**		**355**		**20**		**182**		**654**	**100.00%**
Reported to have slept under a mosquito net											
*Last night, did you sleep under the mosquito net? (Yes/No)*											
In the night preceding the survey (among all workers, *n =* 702)											
All types of nets	Male	50	52.00%	169	46.00%	11	55.00%	101	53.00%	331	49.10%
	Female	47	48.00%	198	54.00%	9	45.00%	89	47.00%	343	50.90%
***Total***		***97***		***367***		***20***		***190***			
In the night preceding the survey (among workers that own ITNs, *n =* 628)											
ITNs	Male	49	51.00%	147	44.00%	11	55.00%	85	50.00%	292	47.00%
	Female	47	49.00%	188	56.00%	9	45.00%	85	50.00%	329	53.00%
***Total***		***96***		***335***		***20***		***170***			
*Over the last 3 nights (including last night) in how many nights did you sleep under the net? (0,1,2,3)*											
All of the last three nights (among all workers, *n =* 702)											
All types of nets	Male	49	51.00%	166	46.00%	11	55.00%	97	53.00%	323	49.00%
	Female	47	49.00%	194	54.00%	9	45.00%	86	47.00%	336	51.00%
***Total***		***96***		***360***		***20***		***183***			
All of the last three nights (among workers who own ITNs, *n =* 628)											
ITNs	Male	48	51.00%	141	43.00%	11	55.00%	84	51.00%	284	46.90%
	Female	47	49.00%	184	57.00%	9	45.00%	82	49.00%	322	53.10%
***Total***		***95***		***325***		***20***		***166***			
*Since arrival to the farm, how often do you usually sleep under the mosquito net? (n* = 702*)*											
Always	Male	49	51.00%	156	40.00%	10	50.00%	96	49.00%	311	44.30%
	Female	47	48.00%	189	48.00%	9	45.00%	86	44.00%	331	47.20%
Often	Male	1	1.00%	12	3.00%	1	5.00%	4	2.00%	18	2.60%
	Female	0	0.00%	11	3.00%	0	0.00%	3	2.00%	14	2.00%
Sometimes	Male	0	0.00%	4	1.00%	0	0.00%	2	1.00%	6	0.90%
	Female	0	0.00%	1	0.00%	0	0.00%	0	0.00%	1	0.10%
Never	Male	0	0.00%	11	3.00%	0	0.00%	2	1.00%	13	1.90%
	Female	0	0.00%	7	2.00%	0	0.00%	1	1.00%	8	1.10%
***Total***		**97**		**391**		**20**		**194**			
Understand benefits of using an insecticide treated bet net (among workers that own ITN, *n =* 628)											
Yes	Male	43	45.00%	136	40.00%	11	55.00%	68	40.00%	258	41.10%
	Female	46	48.00%	169	50.00%	8	40.00%	68	40.00%	291	46.30%
No	Male	6	6.00%	14	4.00%		0.00%	18	11.00%	38	6.10%
	Female	1	1.00%	22	6.00%	1	5.00%	17	10.00%	41	6.50%
***Total***		**96**		**341**		**20**		**171**			
Reported time of sleeping (*n =* 674)											
17:00–17:59 pm		0	0.00%	1	0.00%	0	0.00%	0	0.00%	1	0.10%
18:00–18:59 pm		0	0.00%	17	5.00%	0	0.00%	8	4.00%	25	3.70%
19:00–19:59 pm		19	20.00%	126	34.00%	2	10.00%	46	24.00%	193	28.60%
20:00–20:59 pm		55	57.00%	151	41.00%	13	65.00%	88	46.00%	307	45.50%
21:00–21:59 pm		19	20.00%	54	15.00%	4	20.00%	29	15.00%	106	15.70%
22:00–22:59 pm		4	4.00%	9	2.00%	1	5.00%	18	9.00%	32	4.70%
after 23:00 pm		0	0.00%	9	2.00%	0	0.00%	1	1.00%	10	1.50%
***Total***		***97***		***367***		***20***		***190***			

*LLIN* Long lasting insecticide treated net, *LLIHN* Long lasting insecticide treated hammock net, *ITN* Insecticide treated net

### Willingness to pay and satisfaction with LLIN lending scheme

49% (344/702) of the interviewed farm workers (Table 6) expressed their willingness to pay some money to have their own mosquito net. Although men were 1.4 times more likely to purchase a bed net compared to women, this was not statistically significant. Interestingly, workers who do not own any mosquito nets were more willing to pay (*P =* 0.02131) for mosquito nets than those who own the nets. The average amount they could afford to pay for one mosquito net was about USD4, ranging from a quarter of dollar to over USD12. The mean number of LLINs the farm owners actually had at the time of survey (13 per farm) was significantly lower than the number of LLINs they borrowed from the lending scheme (19 per farm), and 28% of the farm owners reported not having enough LLINs for their workers. 99% of the farm owners expressed their satisfaction with the LLIN lending scheme because they can get free LLINs for their workers protection against malaria. Nearly 98% of the farm workers reported to be satisfied with the way the farm owners lend LLINs to the workers, and 60% of the farm workers reported to have been given some form of verbal message by their respective farm owner on the use of LLINs, where the most consistent messaging (85%) was “sleeping under LLIN every night and everywhere to protect yourself from malaria”. Sharing (re-using) LLINs that have already been used by other workers did not seem to be a major concern. Nets from the lending scheme that were used, dirty and had a bad smell was cited as a main reasons for not using LLINs by only four of the workers in this survey. 17% of the 24 farm workers who did not like using LLINs and less than 1% of all the workers.

**Table 6:** Bed net ownership, use and willingness to pay

	Male (*n =* 348)		Female (*n =* 354)		*OR*	*X*^2^ *P*–value
	Number (%)	*95% CI*	Number (%)	*95% CI*		
Have net_all types	319 (92)	88–94	335 (95)	92–97		1.9804, df = 1 0.1593
Net use last night_all types	331 (95)	92–97	343 (97)	94–98		1.0212, df = 1 0.3122
Willing to pay	184 (53)	47–58	160 (45)	40–51	1.4	3.8360, df = 1 0.5016
Scheme satisfaction	341 (98)	96–99	344 (97)	95–99		0.2073, df = 1 0.6488
	Willing to pay (*n =* 344)		Not willing to pay (*n =* 358)			
	Number (%)	*95% CI*	Number (%)	*95% CI*		
Have net_all types	312 (91)	87–93	342 (95)	93–97		
Net use last night_all types	324 (94)	91–96	350 (98)	95–99		

## Discussion

### Mobility

About half of the workers that responded in this survey reported to be residents of the same or nearby provinces, with an equal representation of gender, majority of who are in the 21–40 year age group, married and with an accompanying family. This demographic was also seen in a separate study (PSI, 2013) among private and family run plantations and in mines that mostly attracted and employed local workers (57%, local people defined as having lived or worked in the area for over one year) with over 70% of workers coming with their families. This survey was conducted over the months of January to February 2013 when there is a peak of migrant workers during the tending seasons, as with the mid-year peak where the planting happens. Although this survey did not detail the nature of the workers occupations over the year, the very short length of stay in the farms of interview (78% of workers with less than 30 days stay) suggests that movements of a majority of these workers follow circular migration trends where after a few weeks of work in a farm or plantation, they move to work in a gold mine, road or a hydro dam construction or engaged in logging activities as forest workers [16]. The use of taxis was the most common (74.5%) means of transportation among the MMPs to travel from their place of origin to the farm. The CAP-Malaria developed malaria information and behaviour change education materials, to be used among 100 taxi drivers and 13 bus companies as a strategy to promote malaria messages among travelers and passengers, of whom many were migrant workers. From this study, it appeared that majority (87.4%) of respondents knew that sleeping under LLINs/LLIHNs protect against malaria even though 77.2% had just primary or no formal education at all. This suggested that the malaria health education strategies used among these workers could be understood regardless of their education background. Ensuring LLIN/LLIHN availability among plantation owners and timing distribution among plantation workers during these seasonal peaks would be a strategic approach in maximizing coverage among mobile populations.

### Access

46.6% of respondents borrowed LLINs/LLIHNs from farm owners. Thus, the lending scheme provided substantial complementary access to the national free-net distribution campaign. CNM has decided on free LLIN distribution to MMPs who stay longer than six months in endemic area and a net lending scheme among those who stay less than 6 months. Given that 91% of MMPs stayed shorter than 6 months in the endemic areas, this finding is important to guide policy formulation on LLIN free distribution or a lending scheme. A more robust method of quantification is needed to determine who among this highly mobile niche group of workers are the most at risk for malaria, their sleeping patterns, net preferences and net durability in plantation settings. In this study, there were 6.8% of workers that didn’t own any nets and 3.7% with untreated nets. These groups could be targeted through close monitoring of farm owners to be proactive in providing LLINs to new comers or those with untreated nets. The routine monitoring by VMW to farm sites under the LLIN “top-up scheme” to ensure optimum net coverage among MMP workers at any point in time while in the farms, should be an area for further evaluation.

### Ownership and utilization

Among the workers who owned LLINs/LLIHNs, the findings suggested very high proportion of use with 99% (621/628) sleeping under LLINs/LLIHNs the night before the survey and 96% (606/628) in the last 3 nights before the survey. There was no significant difference in reported use among males and women. 45% reported sleeping by 8:00–9:00 pm. Although net preference was not part of this study, a separate survey [8] among the plantation and company workers in Koh Kong and Kratie provinces, 69% of the workers slept under a treated net the previous night (20% ITN, 46% LLIN, 3% treated hammock net). An assessment of net condition and quality was not performed in this survey. Future surveys should examine how the program retains nets through the lending scheme given net durability assessments suggests that the life of LLINs in the field may be shorter than the three to five years as recommended by manufacturers, and that net durability is highly variable by location [17, 18].

### Free vs others

This study showed 50% of workers interviewed were willing to purchase a bed net and would suggest an opportunity for the project to possibly introduce subsidized vouchers for LLINs [19]. However, in a separate survey of plantations covering 17 provinces in Cambodia, [12], the majority of enterprises said they would prefer to give nets rather than lend nets. Possible reasons included lent bed nets need washing, counting, dislike of sleeping under a new previously used by someone else, and that the workers are poor and farm managers would prefer to give the nets. In the Greater Mekong Sub-region (GMS) countries today, bed net delivery are provided through a mix of public, NGO and private delivery strategies, As countries attempt to rapidly scale up ITN coverage, they cannot neglect the short- and long-term benefits of using commercial channels as a complement to well-targeted distribution of free or nearly-free nets to populations who do not reside in communities throughout the year. These populations may miss out on annual and sometimes continuous bed net distribution schemes; and will rely more on the private sector channels– formal or informal, and employer or worksite enabled access to malaria prevention commodities. Although there are publications on schemes and programs targeted for populations at risk for malaria through a variety of strategies namely, free distribution through public health facilities [20], commercial sector (e.g., United States Agency for International Development-NetMark Project countries, http://pshi.fhi360.org/whatwedo/projects/netmark.html); free distribution through infant vaccination campaigns [21, 22], social marketing (e.g., in Kenya); and integrated approaches involving free distribution of LLINs through some kind of a voucher scheme, there is not much published on evaluations of bed net delivery schemes for mobile populations in the GMS countries. In this survey, no questions about farm owners’ interest in buying nets for their workers were asked during the assessment but from separate interviews conducted in 2014 [23], there was extremely limited interest in purchasing any nets to protect workers.

### Acceptance by farm owners

This survey found that when farm owners were asked “Are you satisfied with the lending scheme?” all owners interviewed were very satisfied (205/206 farm owners, 99.5%) with the LLIN scheme because they received free nets, which would protect their workers from malaria. However, the monitoring tool/activity developed and conducted by the CAP-Malaria assessed farm owners’ management and lending procedures as well as workers’ knowledge of malaria and difficulties they faced in borrowing nets from farm owners. These monitoring activities revealed that although the scheme distributed a significant number of nets to target populations, the “buy-in” from owners within sites was very limited. In addition, weak management of the lending scheme included lack of record keeping and a lack of accountability in some LLIN buffer stocks [23, 24].

## Conclusions

Large-scale vector control interventions occurred during the last few years in Cambodia particularly based on the free distribution of LLINs. In forest and forest plots settings in Cambodia, evidence suggested [25, 26] that LLIN provided just partial protection against malaria and the importance of additional protective methods in such environments (long-lasting insecticidal hammocks, topical or spatial repellents, etc.) are necessary and can have added value in tackling residual malaria transmission [27, 28]. In this study, LLIN distribution channeled through a lending scheme with farm owners for their workers provides a substantial (46.6%) complementary bed net accessibility to the national free-net distribution campaign thus promoting almost total LLINs coverage and use in remote malaria endemic areas.

The research team recommends further studies on the physical durability and insecticide retention of LLINs and treated materials in plantation settings along with a more in-depth understanding of behavioural practices among MMPs with regards to net choices and user-preferences. These studies would be helpful in informing strategies to enhance LLIN use through appropriate delivery channels and frequency and timing of distribution. Approaches could include extending distribution schemes to involve the commercial sector, through both formal and informal channels, along the mobility pathway from origin, transit and destination [29].

In this study, self-reported ITN usage was found to be very high (92%) among surveyed migrant workers. Although there is potential for social response bias in this survey as MMPs might have felt pressured to respond with the “correct” answer, and thus does not rule out that in some settings, consideration should be given for direct distribution over a lending scheme especially in instances where the lending itself is problematic due to high managerial burden and limited accountability by farm owners. Alternatively, a more targeted incentive strategy for farm owners for their greater buy-in could be an option.

More studies are also needed on the impact of deforestation and development of new plantations; have on farm ecologies, and the bionomics of the vectors in these settings and how these changes the effectiveness of treated nets, insecticide resistance and personal protection methods used in outdoor transmission like treated hammocks nets, treated clothing and temporary shelters, and topical and spatial repellents. This understanding will guide a more efficient targeting of vector control interventions within Cambodia’s malaria micro stratification [30], allowing more innovative approaches to target populations at risk either through universal coverage of vector control or specific top up strategies for residual transmission.

## Additional file

Additional file 1: Multilingual abstracts in the five official working languages of the United Nations. (PDF 384 kb)

## Abbreviations

CNM: Cambodian National Malaria Program
GMS: Greater Mekong Sub-region
ITN: Insecticide treated net
LLIHN: Long lasting insecticide treated hammock net
LLIN: Long lasting insecticide treated net
MEAF: Malaria Elimination Action Framework
MMP: Mobile and migrant population
NGO: Non-governmental organization
PSI: Population Services International
URC: University Research Co. LLC
VMW: Village malaria worker
WHO: World Health Organization

## References

[1] World Health Organization (2007). Insecticide-treated mosquito nets: a WHO position statement.

[2] Binka FN, Indome F, Smith T (1998). Impact of spatial distribution of permethrin-impregnated bed nets on child mortality in rural northern Ghana. Am J Trop Med Hyg.

[3] Hawley WA, Phillips-Howard PA, ter Kuile FO, Terlouw DJ, Vulule JM, Ombok M, Nahlen BL (2003). Community-wide effects of permethrin-treated bed nets on child mortality and malaria morbidity in western Kenya. Am J Trop Med Hyg..

[4] Kilian A, Boulay M, Koenker H, Lynch M (2010). How many mosquito nets are needed to achieve universal coverage? Recommendations for the quantification and allocation of long-lasting insecticidal nets for mass campaigns. Malaria J.

[5] World Health Organization (WHO). Strategy for malaria elimination in the Greater Mekong Subregion: 2015–2030. 2015. World Health Organization Regional Office for the Western Pacific. ISBN 978 92 9061 718 1.

[6] World Health Organization (WHO). World Malaria Report; 2014.

[7] National Institute of Statistics, Directorate general for health, and ICF Macro.2011. Cambodia demographic and health survey 2010. https://dhsprogram.com/pubs/pdf/FR249/FR249.pdf. Accessed 3 Feb 2016.

[8] Partners for Development. Knowledge, attitude and practice survey among plantation and construction workers in malaria areas in Kratie and Koh Kong Provinces. 2015. http://pfd.org/reports-publications/ . Accessed 23 Feb 2016.

[9] Cambodia National Malaria Program, London School of Hygiene and Tropical Medicine and Malaria Consortium. Strategy to address migrant and mobile populations for malaria elimination in Cambodia. 2013.

[10] Partners for Development Cambodia with support from The Global Fund to fight aids, Tuberculosis & Malaria. The Role of Partners for Development in Malaria Prevention and Control in Cambodia, 2004–2014. 2015. http://pfd.org/wp-content/uploads/GF_Desk_Review_2004-2014_Final.pdf. Accessed 5 Feb 2016.

[11] President’s Malaria Initiative (PMI). PMI Greater Mekong Subregion Malaria Operational Plan FY 2014. http://www.pmi.gov/docs/default-source/default-document-library/malaria-operational-plans/fy14/mekong_mop_fy14.pdf?sfvrsn=12.Accessed 8 Feb 2016.

[12] PSI Plantation Survey 2013. Presentation made at the ‘Technical consultation on improving access to malaria control services for migrants and mobile populations in the context of the emergency response to artemisinin resistance in the Greater Mekong Subregion (GMS) 22–23 May 2014 Ha Noi, Viet Nam’. WHO Regional Office for the Western Pacific. 2014. http://www.who.int/malaria/areas/greater_mekong/access-migrants-mobile-populations/en/ Accessed 18 Feb 2016.

[13] Char Meng Chuor. National Center for Malaria, Parasitology Entomology (CNM). Benefits to National Malaria Programs from Regional Support: The Cambodia Case. 2014. http://aplma.org/upload/resource/Papers/Benefits%20to%20National%20Malaria%20Programs%20from%20Regional%20Support%20The%20Cambodia%20case.pdf. Accessed 18 Feb 2016.

[14] University Research Co., LLC. Technical Brief. Malaria Control in Cambodia: Lending scheme for Long-lasting Insecticide Nets to Mobile and Migrant Workers. 2011. http://www.urc-chs.com/resources/malaria-control-cambodia-lending-scheme-long-lasting-insecticide-nets-mobile-and-migrant. Accessed 13 Jan 2016.

[15] USAID. Technical Brief. Malaria control in Cambodia: Lending scheme for long-lasting insecticide nets to mobile and migrant workers. 2011. http://www.urc-chs.com/sites/default/files/MCC_LLIN_loan_brief_final.pdf. Accessed 8 Feb 2016.

[16] Guyant (2015). Malaria and the mobile and migrant population in Cambodia: a population movement framework to inform strategies for malaria control and elimination. Malar J.

[17] President’s Malaria Initiative. Greater Mekong Subregion Malaria Operational Plan FY 2015. https://www.pmi.gov/docs/default-source/default-document-library/malaria-operational-plans/fy-15/fy-2015-greater-mekong-subregion-malaria-operational-plan.pdf?sfvrsn=5. Accessed 20 Feb 2016.

[18] C L, Grabowsky M, McGuire D, deSavigny D (2007). Quick wins versus sustainability: options for the upscaling of insecticide-treated nets. Am J Trop Med Hyg.

[19] Networks project vector control assessment in Greater Mekong Sub region. Review of malaria prevention strategies, tools, stakeholders, target group segmentation, behavioural issues, private sector development options. https://www.malariaconsortium.org/media-downloads/295/Networks%20project%20vector%20control%20assessment%20in%20Greater%20sub-Mekong%20Region. 2012. P.58–59.

[20] Noor Abdisalan M, Mutheu Juliette J, Tatem Andrew J, Hay Simon I, Snow Robert W (2009). Insecticide-treated net coverage in Africa: mapping progress in 2000–07. The Lancet.

[21] Grabowsky M, Nobiya T, Ahun M, Donna R, Lengor M, Zimmerman D (2005). Distributing insecticide-treated bednets during measles vaccination: a low-cost means of achieving high and equitable coverage. Bull World Health Organ.

[22] Centers of Disease Control and Prevention (2005). Distribution of insecticide-treated bednets during an integrated nationwide immunization campaign–Togo, West Africa. MMWR Morb Mortal Wkly Rep.

[23] USAID. Mid-Term Performance Evaluation: Control and Prevention of Malaria (CAP-Malaria) In Burma, Cambodia And Thailand. Cooperative Agreement Number: AID-486-A-12-00001.May 31, 2014. http://pdf.usaid.gov/pdf_docs/PA00JWRP.pdf. Accessed 12 Feb 2016.

[24] USAID. Evaluation of the Malaria Control in Cambodia Project. Final Report, Sept 28th, 2012. Contract no. GHS-I-00-07-00010-00. http://pdf.usaid.gov/pdf_docs/pdacu493.pdf. Accessed 12 Feb 2016.

[25] Sochantha T, Van Bortel W, Savonnaroth S, Marcotty T, Speybroeck N, Coosemans M (2010). Personal protection by long-lasting insecticidal hammocks against the bites of forest malaria vectors. Tropical Med Int Health.

[26] Trung HD, Van BW, Sochantha T, Keokenchanh K, Briët OJT, Coosemans M (2005). Behavioural heterogeneity of Anopheles species in ecologically different localities in Southeast Asia: a challenge for vector control. Tropical Med Int Health.

[27] Gryseels (2015). Re-imagining malaria: heterogeneity of human and mosquito behaviour in relation to residual malaria transmission in Cambodia. Malaria J.

[28] Thang N, Erhart A, Speybroeck N (2009). Long-lasting insecticidal hammocks for controlling forest malaria: a community-based trial in a rural area of Central Vietnam. PLoS One.

[29] World Health Organization. Approaches for mobile and migrant populations in the context of malaria multidrug resistance and malaria elimination in the Greater Mekong Subregion. 2016. http://www.wpro.who.int/mvp/documents/mmp_b5219/en/ . Accessed 10 Feb 2016.

[30] Kolaczinski J (2014). Vector control to eliminate artemisinin resistant malaria in the greater Mekong subregion. Lancet Infect Dis.

