# Supplementary material for: Assessment of net lending strategy to better reach mobile and migrant populations in malaria endemic areas of Cambodia
Source: Infect Dis Poverty. 2018 Oct 18;7:107. doi: 10.1186/s40249-018-0489-1 (PMC6194585; doi:10.1186/s40249-018-0489-1)
Supplement: Supplementary file 1 — Multilingual abstracts in the five official working languages of the United Nations. (PDF 384 kb) [file 40249_2018_489_MOESM1_ESM.pdf]

Translation of the abstract into the six official working languages of the United Nations

تقييم استرتيجية صافي الإقراض لتحسين الوصول إلى السكان الرحل والسكان المهاجرين في المناطق الموبوءة بالملاريا بكمبوديا

Sopheab Heng, Sreng Bun, Chy Say, Nguon Sokomar, ,Dysoley Lek, Deyer Gopinath, Sovann Ek Kheang Soy Ty, Huy Rekol

نبذة مختصرة

معلومات عامة: في كمبوديا، تشمل الهجرة الداخلية انتقال المهاجرون من مناطق غير موبوءة بالملاريا إلى مناطق موبوءة بالملاريا والعكس بالعكس. معظهم يعمل في المزارع أو الغابات بمستويات مختلفة من انتقال الملاريا. في كمبوديا، وكتنفيذ للنهج الوطني لضمان وصول واستخدام الناموسية المشبعة بالمبيدات الحشرية طويلة المفعول (LLIN) بين السكان الرحل والسكان المهاجرين (MMPs)، وتم إطلاقخطة إقراض الناموسيات المشبعة بالمبيدات الحشرية طويلة المفعول بين عمال المزارع. وخلال هذا البرنامج، سيتم توزيع الناموسيات المشبعة بالمبيدات الحشرية طويلة المفعول والناموسيات الأرجوحة المشبعة بالمبيدات الحشرية طويلة المفعول(LLIHNs) سنويًا في محل العمل مثل (المزارع القديمة، والمنشآت الزراعية، والمواقع الصناعية، كما حُددت من جانب موظفيّ مركز الإدارة الصحية) بمعدل ناموسية واحدة لكل عامل. الهدف الرئيس من تلك الدراسة هو تقييم إمكانية السكان الرحل والسكان المهاجرين في الحصول على LLINs من خلال خطة الإقراض وبالتعاون مع مُلاك المنشآت الزراعية في المناطق النائية الموبوءة بالملاريا بكمبوديا.

الأساليب: <bx/> أجري استطلاع مقطعي مجتمعي بين السكان الرحل والسكان المهاجرين باستخدام طريقة (اعتيان المجموعة) على مرحلتَيْن. وكان إطار العينات في أربع مقاطعات وهم بانتي مينشيه، وباتامبانغ، وبيلن، وبورسات في الحدود الغربية والشمالية لكمبوديا مع تايلاند، حيث تم تنفيذ خطة إقراض LLIN ، ومكان عمل ما يقرب من 100000 عامل سنويًا من السكان الرحل والسكان المهاجرين. وأجري التقييم في الفترة من كانون الثاني/يناير إلى شباط/فبراير للعام 2013 في تلك المقاطعات الأربع. وقُدر أنه يلزم توفير 768 عاملًا.

النتائج: وقد تم إجراء مقابلة ما مجموعه 702 من السكان الرحل والسكان المهاجرين. وتبلغ نسبة الذكور الى الإناث ١ الى ١. وكانت المجموعة العمرية من 21-60 هي الأكبر حسابياً بنسبة 77.6%. وحوالي 91% من السكان الرحل والسكان المهاجرين استمروا في التواجد بالمزرعة لمدة أقل من ستة شهور. وأمتلك 93.2% منهم ناموسيات سواء كانت معالجة أو غير معالجة بالمبيدات الحشرية. وتم حساب عدد الناموسيات LLINs وLLIHNs لنسبة 89.5 ؛ووجد أن نسبة 46.6% منهم أقترضوا الناموسيات ضمن برنامج الإقراض. ومن بين أولئك العمال الذين أمتلكوا LLINs وLLIHNs، نام منهم نسبة 99% تحت الناموسيات في الليلة السابقة. وبرغم ذلك، علم نسبة 87.4% ممن ينامون تحت الناموسياتLLINs/LLIHNs إنها تحميهم من الملاريا.

الأستنتاج: تُعدّ خطه إقراض LLIN قناة مهمة لنسبة مستدامة لصافي إمكانية وصول تبلغ (46.6%) بالحملة الوطنية للتوزيع المجاني للناموسيات بكمبوديا في الأماكن النائية الموبوءة بالملاريا.

Translated from English version into Arabic by Mohamed Fouad, proofread by Ruqaya, through

TRANSLATORS
WITHOUT BORDERS

评估长效杀虫剂浸泡蚊帐贷款策略对柬埔寨疟疾流行区流动和移民人口的可及性

Dysoley Lek, Deyer Gopinath, Sovann Ek, Sopheab Heng, Sreng Bun, Chy Say, Nguon Sokomar, Kheang Soy Ty, Huy Rekol

摘要

引言：柬埔寨国内移民包括从疟疾非流行区迁移到流行区以及反方向迁移的人群。大部分移民在不同疟疾流行程度的农场或森林工作。在柬埔寨，作为确保移动和移民人口（MMPs）中长效杀虫剂浸泡蚊帐（LLINs）可及性和使用的国家疟疾防治策略之一，在农场工人中开始实施LLINs 贷款计划。通过该贷款计划，LLINs 和长效杀虫剂处理吊床网（LLIHNs）每年将按比例（即每位工人 1 个 LLIN）分发至各工作场所（如由经营区和卫生中心工作人员核实的长期农场、种植园、工业区）。本研究的主要目的是通过柬埔寨偏远疟疾流行区的种植园主开展贷款计划，评估 MMPs 对 LLINs 的可及性。

方法：本研究采用两阶段整群抽样法对 MMPs 进行横断面调查。抽样范围是柬埔寨西部和西北部与泰国接壤的班提米尼奇、马德望、拜林和普萨特 4 省的农场。LLIN 贷款计划在上述 4 省实施，这些地区每年约 10 万名 MMPs。于 2013 年 1～2 月开展评估，预估将涉及 768 名工人。

结果：共调查 702 名 MMPs，男女比例为 1:1。21～60 岁年龄组占比最大，为 77.6％。约 91％ MMPs 在农场的工作时间少于 6 个月，其中，93.2%的人拥有未经处理或经杀虫剂处理的蚊帐。拥有 LLINs 和 LLIHNs 的人占 89.5%，其中 46.6%从贷款计划获得。在拥有 LLINs/LLIHNs 的工人中，99％的人前一天晚上睡在 LLINs/LLIHNs 中。但是，仅 87.4%的人知道睡在 LLINs/LLIHNs 中可预防疟疾。

结论：LLIN 贷款计划为柬埔寨偏远疟疾流行区的免费蚊帐分配活动提供了重要的交付渠道，使 MMPs 对 LLINs 的可及性达到 46.6％。

Translated from English version into Chinese by Fan Yang, edited by Pin Yang

Évaluation de la stratégie de prêt de moustiquaires dans le but de mieux atteindre les populations mobiles et migrantes des régions du Cambodge où le paludisme est endémique

Dysoley Lek, Deyer Gopinath, Sovann Ek, Sopheab Heng, Sreng Bun, Chy Say, Nguon Sokomar, Kheang Soy Ty, Huy Rekol

Résumé

Contexte: Au Cambodge, les migrations internes peuvent aller de zones où le paludisme n’est pas endémique vers des zones d’endémie et vice-versa. La majorité de ces migrants travaillent dans des exploitations agricoles ou des forêts où le niveau de transmission du paludisme est variable. Un programme de prêt de moustiquaires imprégnées d’insecticide de longue durée (MILD) a été mis en place à destination des populations des ouvriers agricoles au Cambodge, parmi d’autres initiatives nationales visant à assurer la disponibilité et l’utilisation des MILD parmi les populations mobiles et migrantes. Dans le cadre de ce programme de prêt de moustiquaires, des MILD et des hamacs imprégnées d’insecticide de longue durée (HILD) seront distribués chaque année sur les lieux de travail (par ex. exploitations agricoles permanentes, plantations et sites industriels, identifiés par le personnel du district opérationnel et du centre de santé) à raison d’une MILD par ouvrier. L’objectif principal de cette étude est d’évaluer l’accès des populations mobiles aux MILD, par l’intermédiaire d’un programme de prêt

organisé auprès des propriétaires de plantations dans des régions reculées du Cambodge où le paludisme est endémique.
Méthodes: Cette étude a été réalisée sous la forme d'une enquête transversale menée parmi les PMM en utilisant la méthode d'échantillonnage en grappes à deux niveaux. La base d'échantillonnage était la liste des exploitations agricoles des quatre provinces de Banteay Meanchey, Battambang, Pailin et Pursat, à l'ouest et au nord-ouest du Cambodge, à la frontière avec la Thaïlande, où le programme de prêt de MILD a été mis en place et où l'on estime que 100 000 PMM travaillent chaque année. Cette évaluation a été réalisée de janvier à février 2013 dans ces quatre provinces. On a estimé que l'échantillon devrait inclure 768 ouvriers agricoles.
Résultats: Au total, 702 PMM ont été interrogées. Le ratio homme/femme est de 1:1. Le groupe des 21-60 ans constitue la classe d'âge la plus nombreuse, avec 77,6 % des sujets. Environ 91 % des PMM sont restées sur l'exploitation pendant moins de six mois. 93,2 % de ces populations possédaient des moustiquaires non traitées ou imprégnées d'insecticide. Les MILD et les MHILD représentaient 89,5 % de ce chiffre et 46,6 % avaient été empruntées dans le cadre d'un programme de prêt. Parmi les ouvriers possédant un MILD/MHILD, 99 % avaient dormi sous une MILD/MHILD la nuit précédente. Pourtant, seuls 87,4 % d'entres eux savaient que dormir sous une MILD/MHILD les protégeait du paludisme.
Conclusions: Le programme de prêt de MILD représente un réseau de distribution important et permet un pourcentage conséquent d'accès aux moustiquaire (46,6 %) dans le cadre de la campagne nationale cambodgienne de distribution de moustiquaires gratuites dans les régions reculées où le paludisme est endémique.

Translated from English version into French by Marine Pic, proofread by Suzanne Assenat, through

TRANSLATORS
WITHOUT BORDERS

Оценка стратегии чистого финансирования с целью увеличить охват мигрантов и мобильного населения в эндемичных по малярии районах Камбоджа

Dysoley Lek, Deyer Gopinath, Sovann Ek, Sopheab Heng, Sreng Bun, Chy Say, Nguon Sokomar, Kheang Soy Ty, Huy Rekol

Краткий обзор

Предпосылки: В Камбодже внутренняя миграция включает людей, перемещающихся из неэндемичных по малярии районов в эндемичные по малярии районы и наоборот. Большинство из этих людей работают на фермах или занимаются лесохозяйственными работами, где наблюдаются различные уровни передачи малярии. В Камбодже среди людей, занятых лесохозяйственными работами, в качестве схемы финансирования противомоскитными сетками, обработанных инсектицидом длительного действия (СОИД), началось осуществление одного из национальных подходов по обеспечению доступа СОИД чтобы использовать их среди мигрантов и мобильного населения MMPs (mobile and migrant populations - MMPs). Благодаря программе

чистого финансирования, СОИД и подвесные СОИД, будут распространяться ежегодно на рабочих местах (например на многолетних фермах, на плантациях, на производственных площадках, как определено оперативным округом и персоналом медицинской организации) в соотношении одна противомоскитная сетка на одного рабочего. Главная цель данного исследования — оценка доступности СОИД для MMPs с помощью схемы финансирования для владельцев плантаций в отдаленных в эндемичных по малярии районах в Камбодже.

Методы: Исследование проводилось методом перекрестного обследования среди MMPs используя двухступенчатую кластерную выборку. Выборочная совокупность — это список ферм, находящихся в четырех провинциях, таких, как Бантеймеантьей, Баттамбанг, Пайлин и Поусат в западной и северо-западной Камбодже на границе с Таиландом, где была выполнена схема финансирования СОИД, и по оценкам работали ежегодно 100 000 MMPs. Исследование проводилось с января по февраль 2013 года в этих четырех провинциях. По оценкам, 768 рабочих должно было быть задействовано в исследовании.

Результаты: В общей сложности был проведен опрос 702 MMP. Соотношение мужчин и женщин составило 1:1. Возрастная группа 21–60 лет была самой большой и составляла 77,6 %. Около 91 % MMP находились на ферме менее 6 месяцев. Из них 93,2 % имели необработанные или обработанные инсектицидами сетки. Обычные и подвесные москитные сетки составили 89,5 %, принимая во внимание, что 46,6 % сеток было приобретено за счет схемы финансирования. Из рабочих, у которых есть СОИД или подвесные СОИД, 99 % спали под противомоскитными сетками ночью, перед опросом. Однако, только 87,4 % знали, что во время сна сетки СОИД или подвесные СОИД защищают от малярии.

Выводы: Схема финансирования СОИД представляет собой важный канал доставки значительной доли доступности сеток (46,6 %) в Камбоджийской национальной кампании по бесплатному распределению противомоскитных сеток в отдаленных в эндемичных по малярии районах.

Translated from English version into Russian by Alexander Vareiko, proofread by Olga Sharpe, through

TRANSLATORS WITHOUT BORDERS

Evaluación de la estrategia de préstamo de mosquiteros para un mejor alcance a las poblaciones móviles y migrantes en las zonas de paludismo endémico en Camboya.

Dysoley Lek, Deyer Gopinath, Sovann Ek, Sopheab Heng, Sreng Bun, Chy Say, Nguon Sokomar, Kheang Soy Ty, Huy Rekol

Resumen

Contexto: En Camboya, la migración interna implica migrantes que se desplazan de las zonas sin paludismo endémico a las zonas de paludismo endémico y viceversa. La mayoría de ellos trabaja en granjas o bosques con varios niveles de transmisión de paludismo. En Camboya, como parte de las

acciones nacionales para garantizar la accesibilidad y el uso de mosquiteros tratados con insecticidas de larga duración (LLIN, por sus siglas en inglés) entre las poblaciones móviles y migrantes (MMP, por sus siglas en inglés), se inició un programa de préstamo de LLIN entre los trabajadores de las granjas. Mediante este programa de préstamo, los LLIN y los mosquiteros para hamacas tratados con insecticidas de larga duración (LLIHN, por sus siglas en inglés) se distribuirán anualmente en los lugares de trabajo (por ejemplo, granjas antiguas, plantaciones, emplazamientos industriales, según los haya identificado el personal operacional de distrito y de centros de salud), a razón de un LLIN por cada trabajador. El objetivo principal de este estudio es evaluar la accesibilidad de las MMP a los LLIN mediante un programa de préstamo con los propietarios de plantaciones en las zonas remotas de paludismo endémico en Camboya.

Métodos: El estudio hizo uso de una encuesta transversal entre las MMP con el método de muestreo por conglomerados en dos etapas. El marco de muestreo es la lista de granjas en las cuatro provincias de Banteay Meanchey, Battambang, Pailin y Pursat, al oeste y noroeste de Camboya, limítrofes con Tailandia, donde se implementó el programa de préstamo de LLIN y donde se estima que 100 000 MMP trabajaban anualmente. El período que abarcó la evaluación fue de enero a febrero de 2013 en estas cuatro provincias. Se calculó que se necesitarían 768 trabajadores.

Resultados: Se entrevistaron en total 702 MMP. La razón de hombres:mujeres es 1:1. El grupo de edad de 21-60 años fue el más grande, representando el 77,6 %. Aproximadamente el 91 % de las MMP permaneció en la granja durante menos de seis meses. Un 93,2 % de ellos era dueño de mosquiteros tratados con insecticidas o sin tratar. Los LLIN y LLIHN representaron un 89,5 % y un 46,6 % de ellos tomaron los mosquiteros prestados de un programa de préstamos. Entre los trabajadores que tienen LLIN/LLIHN, un 99 % durmió bajo LLIN/LLIHN la noche anterior. Sin embargo, solo un 87,4 % sabía que dormir bajo LLIN/LLIHN los protege contra el paludismo.

Conclusiones: el programa de préstamo de LLIN proporciona un importante canal de suministro para un considerable porcentaje de accesibilidad a mosquiteros (un 46,6 %) a la campaña nacional camboyana de distribución gratuita de mosquiteros en zonas remotas de paludismo endémico.

Translated from English version into Spanish by A.P. Castaneda, proofread by Sara Fairen, through

TRANSLATORS
WITHOUT BORDERS
